# Angry and “productive”: engagement in gamified precarious work

**DOI:** 10.3389/fpsyg.2026.1833833

**Published:** 2026-08-07

**Authors:** Irina Dzero

**Affiliations:** MCLS, Kent State University, Kent, OH, United States

**Keywords:** affective engagement, algorithmic labor, bonded participation, gamification, gig work, precarious work

## Abstract

We examine the affective engagement of workers in app-mediated food delivery who continue working while explicitly describing the work as harming them financially, emotionally, and bodily, while using the language of betrayed intimacy. Using a mixed-methods design that combines manual coding with eight validated computational instruments and a study-specific linguistic analysis across two corpora—1,385 Glassdoor employer reviews and 1,075 Reddit posts—we identify a sizable minority (*n* = 338, 34.4% of current-employee reviews) of conflicted but persisting workers. On the extended Moral Foundations Dictionary, fairness and authority cross from positive valence in Group A into violation in Group B, while loyalty—the foundation that would have to invert for the bond to break—remains positively valenced in both groups despite a significant decline. These workers avoid saying “I” to shield themselves from the reality of working without sufficient compensation or at a loss; supplicate companies to pay enough so they could continue working; and articulate the platform’s design as betrayal and violation (“abuse machine”). On Reddit, workers describe themselves as addicted (11.5% of posts), report hiding their app use from family, and describe the job design with casino, Pavlov, and Skinner metaphors. We interpret this emotional profile through Patrick Carnes’s framework of the betrayal bond as bonded participation: affective engagement that the gamified iteration of precarious work appears to elicit and sustain. Building upon Shoshana Zuboff’s concept of behavioral surplus, we argue that workers’ outrage, compulsion, and shame are the affective surplus that the platforms convert into continuous productivity, marking a new frontier in the precarization of work.

## 1 Introduction

The stunning success of gig food delivery platforms was made possible by their drivers. DoorDash’s revenue reached $10.7 billion in 2024, a tenfold increase over 5 years (Beyrouthy, 2024); Uber Eats posted $12 billion (Uber Investor, 2025); Instacart $3.3 billion (Haleem, 2024). Yet the median platform worker earns $5.12 per hour after expenses (Human Rights Watch, 2025); 75% of surveyed workers report struggling to afford housing; one-third of DoorDash jobs pay less than $0 after gas and vehicle costs (Working Washington, 2020). Unsurprisingly, the majority of new recruits leave the platforms shortly after signing up. Still, the platforms are growing because they somehow retain the one-third of workers who work 40 + hours per week and perform most of the labor (Parrott and Reich, 2020; Anderson et al., 2021; Glasner, 2020). Gig drivers are often unaware of how little they actually make and refuse to believe the net-earnings numbers when informed (McCullough et al., 2022; Dubal, 2023; Jacobs et al., 2024).

Existing accounts of platform-labor motivation, drawing on Ryan and Deci’s self-determination theory, argue that workers can derive genuine intrinsic satisfaction from the work’s autonomy, competence, and relatedness affordances (Zaman, 2020; Behl et al., 2021). Other studies contest this optimistic reading, arguing that platforms merely simulate the satisfaction of these needs (Gagné et al., 2022) while algorithmic monitoring degrades autonomy in practice (Norlander et al., 2021; Walker et al., 2021; Dubal, 2023; Teachout, 2023). Neither account explains the specific affective signature we observe in our data: workers who explicitly describe the work as abusive, manipulative, and financially harmful—and supplicate the company to pay them a little more so they can continue working. When speaking to peers on Reddit rather than to the employer, they reach for the vocabulary of betrayed intimacy—“Stockholm syndrome,” “I’m a victim of this app’s power over me,” “I’m like an abuse victim who keeps coming back.” When someone offers rational advice to quit because no one is really making them stay, these workers quietly downvote such advice, *without replying*.

In this study, we examine the affective engagement of these workers across two corpora—1,385 Glassdoor employer reviews from DoorDash, Uber Eats, and Instacart, and 1,075 Reddit posts in which workers discuss their inability to quit despite perceived harm. Using a mixed-methods design that combines manual coding with eight validated computational instruments and four study-specific linguistic analyses, we propose to interpret their staying-despite-harm through Carnes (1997) framework of the betrayal bond as bonded participation—affective engagement that the gamified design architecture appears to elicit and convert into continuous productivity. The paper proceeds as follows. Section 2 explains how companies advertise the job as the promise to provide a unique job to special individuals, and then impose unrealistic demands on workers to accept unprofitable offers, betraying that promise and eliciting anger and loyalty as affective surplus which is then converted into continuous productivity. Section 3 describes our methods. Section 4 reports findings across the eight instruments and the language-specific analyses. Section 5 develops the bonded participation framework. Sections 6–8 discuss theoretical implications, limitations, and conclusions.

## 2 Background

Bonded participation begins with a promise. The betrayal bond (Carnes, 1997) can arise in a professional (with an employer, a priest, a therapist), or an intimate context (with a parent or a romantic partner). This relational pattern begins with an asymmetrically powerful party extending a promise of ultimate validation. After this initial period, known as “honeymoon,” the abusive party grows cold, imposes impossible demands, and attacks the victim, for not meeting them. The victim grows sad and confused over the betrayed promise, while the abuser grows preoccupied that the victim may leave and renews the promise. The victim trusts the promise again and becomes euphoric. The honeymoon and betrayal occur at predictable intervals, as a cycle, so the bonded party cannot exit, experiencing shame and denying the reality of the relationship. Carnes terms this staying against self-interest “insane loyalty” (1997: 16). In the context of gig work, such loyalty has a concrete outcome—a lot of labor for the company. The following representative comment from a long-tenured Instacart worker reveals the entire mechanism—the Promise, the betrayal of the Promise, and the conversion of the emotions of pleasure, outrage, and shame into work:

*6000 batches, my neck is broken and gamification running in adrenaline has left me exhausted. The hype is gone and work that I loved is all forgotten except the celebration cards that pop up after each batch telling me I’m a winner when I feel like a loser in my car making this company and its investors rich on my gas and experience that only get acknowledged by tips that have evaporated like the “good” orders.*

This worker was attracted by the Promise (“the hype,” “celebration cards telling me I’m a winner”) and experienced intense enjoyment (“adrenaline,” “loved”). Now that the Promise was betrayed (“the hype is gone”), she feels ashamed (“I feel like a loser”). She also feels exhausted (“my neck is broken”), because she continued working intensively even after compensation “evaporated,” and her labor and resources (“my gas and experience”) went to enrich the company and its investors. Completing each “batch” requires watching the phone to catch an order before others, finding all items on the customer’s list at the store, checking them out, loading them into the car, driving them to the customer’s home, unloading and delivering them, and driving back to catch the next assignment. This worker completed 6,000 such assignments, even though they were not called “work” or paid as such. What was the promise that this worker feels was betrayed?

### 2.1 The promise: work as nonwork, community service, validation, and fun

In their marketing materials, companies promote this job as different from all other jobs and invite future workers to become special through it: “Life as an Uber driver/partner is whatever you want it to be as you are your own boss”; “You call the shots”; “Your schedule, your terms”; “Be Your Own Boss.” By signing up, one becomes a smart entrepreneur: “Turning an everyday chore into an independent business venture where you call the shots.” The recruit will make unique contributions: “Instacart values the unique contribution of every shopper who steps up for their community.” Downloading the app puts one on the path to have an impact on the “world” by joining “those who move the world forward.” In any case, the impact on the “community” is virtually guaranteed: “Shoppers are the heart of the Instacart community, providing an invaluable service to their neighbors.” The work is so special that it is almost never even called work proper. Assignments are renamed “dashes,” “batches,” “rides,” and future recruits are called anything but workers: “dashers,” “shoppers,” “partners,” etc.

By reframing work as opportunity, community service, and freedom, companies also reframe the expectation of compensation—we demand to be paid for “work,” but can we make the same demand for an “earning opportunity,” a “dash,” or a “batch”? Platforms also promise fun: “earn rewards” and “unlock tiers” (Gold, Platinum, and Diamond) to access “exclusive perks designed just for you, because you deserve it.” Commercials for these apps make clear that they are very similar to location-based videogames. In one DoorDash ad, a young female gamer receives a delivery mission on her phone and rushes out to complete it, jumping into her computer screen and becoming a character of the game she was playing. The work is presented as nonwork—fun and free time.

### 2.2 The promise: work as the luxury of leisure

These promises resonate verbatim in the employer reviews. Work that is somehow freedom, when it is truly such in real life, is the mark of high status, affluence, and prestige. As one worker puts it sententiously, “Freedom. One thing even the rich cannot buy is time. Time is important, time with my family, time for myself, time for my responsibilities. Not being stuck at a 9–5 and not having a boss is the best.” The writer ends by decrying low pay, but her authoritative, even didactic tone reveals a deep need for respect and validation—exactly what the platforms target. As Jean Baudrillard noted, the perceived right to leisure is associated with affluence and it has become a great democratic equalizer in consumer society. Baudrillard cited an ad for the tour operator Club Med in which two men bond over fishing and Samoan wine only to discover that they work at the same company back home, one as a manager, and the other as a night watchman (1998: 151). Marketing this work-nonwork as something prestigious available only to the select few promises to bring the luxury of freedom and autonomy to the masses. “Because you deserve it” from the Instacart marketing materials quoted above is a calque of “Because you are worth it,” the slogan of the French cosmetics company L’Oréal, which markets its products as luxuries uniquely available to ordinary consumers.

### 2.3 The promise betrayed: unrealistic demands and the betrayal bond

The apps promise to satisfy the basic human needs for autonomy, competence, and relatedness identified by Ryan and Deci’s self-determination theory (Ryan and Deci, 2017), and even the needs for fun and power, identified by Glasser’s choice theory (Glasser, 1998). Many workers comment that the work is awesome, unique, and great, that they can drive around and listen to podcasts, study, meet people, and explore the neighborhood. This led some scholars to say that workers are having “a whale of a time” (Zaman, 2020) which results in high job satisfaction and productivity (Behl et al., 2021). But all these things can be done without the app—its promise is that beyond the freedom, fun, and relaxation it will bring income. The reality is that after an initial period of receiving lucrative work assignments, known as the “honeymoon” in the driver community (which corresponds exactly to the “honeymoon” in Carnes’s betrayal bond framework), drivers begin receiving mostly unprofitable assignments. To maintain “Diamond-level Priority Access”—that is, to be able to receive offers and use the app intensively—drivers are required to accept at least 70% of jobs, many of which do not even pay for gas, never mind vehicle expenses or unpaid time spent waiting (Rosenblat and Stark, 2016; Dubal, 2023; Working Washington, 2020; Human Rights Watch, 2025). These unrealistic demands provoke intense sentiments of betrayal: outrage, disappointment, and sadness, as we will show in our analysis of the employer reviews. But the intermittent reward mechanism (Dubal, 2023; Teachout, 2023)—the cycle of a well-paying assignment appearing after completing many unprofitable ones—keeps them working. Workers explain this mechanism to each other and feel shame and powerlessness over their continued participation in what they themselves call abuse, as we will now show.

**Figure 1:**
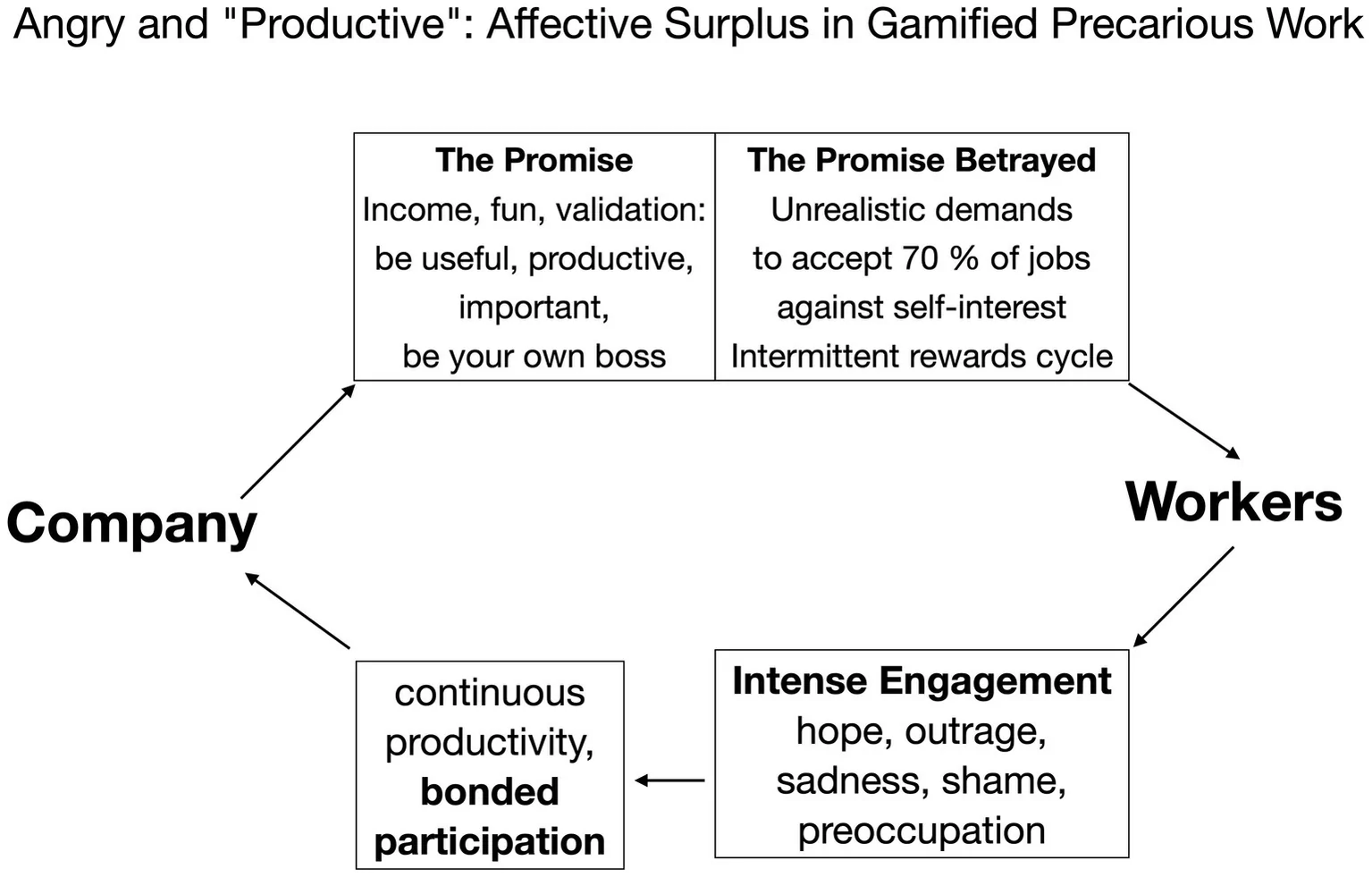
Bonded participation in gamified precarious work.

In this new, gamified iteration of precarious work, the affective surplus of pleasure, outrage, and shame results in continuous productivity (Figure 1). This affective engagement can best be described as bonded participation.

## 3 Materials and methods

### 3.1 Design and analytical pipeline

The engagement of drivers who continue to work voluntarily while describing the work as harming them cannot be identified with off-the-shelf classifiers. We therefore adopted a mixed-methods design and proceeded in six stages:

1. Data collection: Web scraping with metadata retention yielded 1,385 Glassdoor employer reviews and 1,075 Reddit posts.

2. Eligibility filter: Employment-status metadata on Glassdoor reviews yielded 982 current-employee reviews.

3. Manual coding: Two raters independently coded each current-employee review and reconciled to 97% agreement (Cohen’s *κ* = 0.93), yielding Group A (unconflicted engagement, *n* = 644) and Group B (intensely conflicted engagement, *n* = 338).

4. Computational scoring: Each text was scored with eight validated computational instruments and four study-specific linguistic analyses, plus surface stylistic features (text length, exclamations, question marks, capitalization, ellipses).

5. Statistical analysis: Group differences were tested with Welch’s t, χ^2^, Cohen’s d, risk ratios, and *φ* (95% CIs), with Benjamini–Hochberg FDR correction across the test family.

6. Mixed-methods integration: The convergent computational profile was integrated with exemplar quotes drawn from the coded texts and interpreted against Carnes (1997) framework of the betrayal bond.

### 3.2 Corpora

We analyzed two corpora of worker-authored text. The first is employer reviews of three pay-to-work delivery platforms—DoorDash (*n* = 1,002), Instacart (*n* = 173), and Uber Eats (*n* = 210)—posted on Glassdoor from 2023 to 2025; retained metadata include star rating (1–5), employment status, and tenure. The second is 1,075 posts from 17 threads on r/doordash, r/doordash_drivers, r/UberEATS, and r/InstacartShoppers in which workers discuss why they continue to engage with the apps despite perceived harm. At least 580 named users plus an unknown number of deleted accounts participated. Workers in these threads use the confessional register rather than the prosecutorial and moral-injury register that characterizes the employer reviews. Threads were located by topical query; we did not filter on tone.

### 3.3 Manual coding: identifying conflicted engagement in employer reviews

Group assignment was the foundational analytic step. It was motivated by an observation in the corpus of a distinct affective signature—workers who explicitly described the work as harmful yet remained engaged. From the eligible pool of current-employee reviews for DoorDash, Instacart, and UberEats (*n* = 982), we identified this focal group (Group B, intense and conflicted engagement) by a single inclusion criterion: the worker remarks that the costs of participating—gas, vehicle wear, and related expenses—meet or exceed the pay, or that pay is too low to cover them, and does so with discernible emphasis or emotion (evaluative or affective wording, intensifiers, capitalization, exclamations, rhetorical questions, or repetition). A review was excluded from Group B when it did not raise the cost of participation as exceeding the return, or framed losses as occasional, hedged with qualifiers such as “sometimes” or “can be.” Current-employee reviews meeting none of these criteria— focusing their discontent, if any, on customers, app malfunction, and other issues—constituted Group A (unconflicted engagement), the comparison baseline against which the Group B affective signature is measured.

Two raters read every current-employee review and coded it independently, meeting at regular intervals to align their application of the criteria; at final reconciliation they coincided on 97% of reviews (Cohen’s *κ* = 0.93; Landis and Koch, 1977: “almost perfect”), with the remainder resolved by discussion to consensus. The full codebook with anchor examples is in Appendix A. Across the three platforms, one in three current-employee reviews (34.4%) was coded as conflicted (Group B), aligning with prior estimates that approximately one-third of gig drivers perform most of the labor—working more than 32 hours per week and completing 55 percent of all trips (Parrott and Reich, 2020; Anderson et al., 2021; Glasner, 2020). Table 1 reports the funnel by platform.

**Table 1:** Manual coding funnel by platform.

Platform	Exited (former)	Unconflicted (Group A)	Conflicted (Group B)	Group B % of current	Total coded
DoorDash	272 (27.1%)	495 (49.4%)	235 (23.5%)	32.2%	1,002
Instacart	49 (28.3%)	71 (41.0%)	53 (30.6%)	42.7%	173
Uber Eats	82 (39.0%)	78 (37.1%)	50 (23.8%)	39.1%	210
Total	403 (29.1%)	644 (46.5%)	338 (24.4%)	34.4%	1,385

### 3.4 Computational instruments

Every text was scored with eight validated computational instruments. Sentiment was classified with a RoBERTa model trained on social-media text (Barbieri et al., 2020) and with VADER (Hutto and Gilbert, 2014). Discrete emotion was classified with two transformer models—a seven-category model (Hartmann, 2022) and the 28-label GoEmotions model (Demszky et al., 2020). Psycholinguistic content was measured with LIWC-22 (Boyd et al., 2022). Graded emotional intensity was measured with the NRC Emotion Intensity Lexicon (Mohammad, 2018a). Valence, arousal, and dominance were measured with the NRC-VAD lexicon (Mohammad, 2018b). Moral content was measured with the extended Moral Foundations Dictionary (eMFD; Hopp et al., 2021); for each foundation we extracted both a probability and a sentiment score, treating sentiment as primary because probability scales with text length. The instruments are mutually independent, drawn from different training data and methodologies.

### 3.5 Study-specific linguistic analyses

Four lexicons and rule-based measures operationalize constructs central to the framework of the betrayal bond (Carnes, 1997) that the validated instruments do not capture. All four were applied to all three corpora at per-1,000-word density and per-text prevalence; full word lists, regex patterns, and audit results appear in Appendices B–D. Surface stylistic features—per-text rates of exclamations, question marks, capitalization, ellipses, and text length—were tabulated directly.

*Vocabulary of abuse.* A lexicon of nine sub-categories—abuse and mistreatment, exploitation, punishment, threat and coercion, victimization, cheating and theft, manipulation and deception, betrayed promises, and feeling used—capturing the language at the heart of Carnes’s framework. Negative-context rules filter false positives (notably “heart attack,” “car abuse”).

*Avoiding the “I” to hide participation from the self.* We investigated the pattern of shifting away from “I” to shield oneself from painful realities (Orvell et al., 2017, 2019), to demonstrate that speakers normalize and hide from themselves continued participation in harm (Carnes, 1997; Herman, 1992; Brown, 2012). The markers are: generic “you”; “people”; generic role labels (drivers, dashers, shoppers); and null-subject constructions (“Driving 12 h for $60”).

*Supplications and ultimatums.* Company-directed petitions, classified by syntactic construction (direct imperative, please-soft directive, modal demand, embedded passive) and semantic category (financial, technical/algorithmic, humanization). Supplication markers (please, if you can, kindly, help us, need to live) and a contrasting category of ultimatum and exit-threat markers (I quit, we are leaving, you’ll lose drivers) operationalize Carnes’s (1997) account of bonded negotiation—petitioning from inside the bond rather than threatening exit.

*Compulsion.* A lexicon of five subcategories—addiction and dependency, cannot-stop and cannot-quit constructions, cognitive obsession, temporal saturation, and sucked-in / trapped—motivated by Carnes’s (1997) identification of preoccupation and obsession as a sustaining feature of the betrayal bond.

## 4 Results

Group A reviews, Group B reviews, and Reddit posts were analyzed with the same instrument battery and the same study-specific linguistic measures. Table 2 reports findings from the eight validated computational instruments; Table 3 reports findings from the four study-specific linguistic analyses. Both tables provide effect sizes for Group B vs. Group A and for Reddit vs. Group A. Full descriptive statistics, test statistics with 95% confidence intervals, and Benjamini–Hochberg adjusted *p*-values are in Supplementary Tables S1, S2.

**Table 2:** Findings from validated computational instruments.

Measure	Group A	Group B	Reddit	B vs A	R vs A
Sentiment and overall polarity					
RoBERTa sentiment, % negative / % positive	10% / 41%	38% / 17%	32% / 17%	RR = 3.67× / 0.42×***	RR = 4.0×***
VADER compound (M)	+0.30	+0.01	+0.23	d = −0.58***	d = −0.88***
Strongly negative texts (compound ≤ −0.50)	6.2%	24.0%	9.4%	RR = 3.86×***	RR = 2.6×***
LIWC Tone	76.1	52.5	54.8	d = −0.70***	d = −0.70***
Mean star rating (review corpora only)	3.70	2.62	—	d = −0.88***	—
***Affective dimensions: valence, dominance***					
NRC-VAD valence	+0.18	+0.14	+0.12	d = −0.50***	d = −0.90***
NRC-VAD dominance	+0.09	+0.09	+0.04	d = −0.10 (n.s.)	d ≈ −0.54 (approx.)
LIWC power (control / authority topic)	3.64	3.79	0.79	d = +0.04 (n.s.)	d = −0.96***
***Discrete emotions***					
RoBERTa-7 % sadness / anger / disgust / fear	4.5% / 3.0% / 2.0% / 1.9%	8.0% / 9.8% / 5.0% / 3.8%	8.9% / 6.5% / 6.8% / 3.6%	RR = 1.8 to 3.3×***	RR ≈ 2×
GoEmotions % annoyance / disapproval	1.9% / 0.6%	5.3% / 2.1%	4.0% / 2.6%	RR = 2.86× / 3.33×***	RR ≈ 2–4×
NRC intensity: anger / fear / sadness / disgust	0.09 / 0.09 / 0.13 / 0.07	0.15 / 0.19 / 0.22 / 0.16	0.14 / 0.13 / 0.14 / 0.11	d = +0.40 to +0.52***	d ≈ +0.3 to +0.4
LIWC emo_anger (word-count)	0.07	0.06	0.19	d = −0.03 (n.s.)	d = +0.13***
Positive emotions, % Hartmann joy / GoEmotions admiration	30.6% / 16.0%	15.7% / 4.1%	—	RR = 0.51× / 0.26×***	—
***Moral foundations (eMFD; density-per-100 sentiment; negative = violation)***					
Care / Fairness / Authority sentiment	−0.33 / +0.52 / +0.34	−1.12 / −0.51 / −0.91	−1.16 / +0.24 / +0.13	d = −0.28 to −0.45***	d = −0.05 to −0.22**
Loyalty sentiment	+1.70	+0.50	+0.73	d = −0.41***	d = −0.27***
***Us-vs-them framing (LIWC)***					
Pronouns, us-vs-them, we / they	0.06 / 0.42	0.23 / 0.80	0.31 / 0.38	d = +0.25 / +0.27**	d = +0.17 / +0.03
***Bodily and mental toll (LIWC)***					
LIWC health, % of texts	4.3%	6.5%	21.5%	RR = 1.5× (n.s.)	RR = 5.0×***
LIWC mental health, % of texts	0.1%	0.3%	14.6%	RR ≈ 3× (small counts)	RR ≈ 146×***
LIWC physical (M)	0.69	0.79	1.87	d = +0.05 (n.s.)	d = +0.37***
LIWC substances vocabulary	0.004	0.006	0.088	d ≈ +0.02 (n.s.)	d ≈ +1.5

**Table 3:** Findings from study-specific linguistic analyses. Group A (*n* = 644), Group B (*n* = 338), and Reddit (*n* = 1,075).

Measure	Group A	Group B	Reddits	B vs A	R vs A
***Betrayal-frame language (lexicon)***					
Betrayal-frame language, % of texts	1.5%	5.7%	0.37%	RR = 3.80×***	lower than A
Mistreatment vocabulary, combined	4.3%	13.9%	0.74%	RR = 3.24× (φ = 0.17)***	lower than A
– Abuse / mistreat	0.44%	2.37%	0.09%	RR = 5.33×**	RR = 0.21× (n.s.)
– Punish / penalize	1.78%	7.40%	0.0%	RR = 4.16×***	absent
– Cheat / scam / rob	1.48%	4.73%	0.28%	RR = 3.20×**	lower
– Threat / coerce	0.30%	1.48%	0.09%	RR = 4.99×*	lower
***Strategies to avoid saying “I”***					
Combined denial cluster, % of texts	28.9%	45.0%	16.3%	RR = 1.56× (φ = 0.16)***	RR = 0.56× (reverses)
– Generic “you” as proxy for “I”	15.3%	26.9%	3.0%	RR = 1.76×	RR = 0.20×
– Generic “people” as proxy for “I”	3.7%	7.7%	6.1%	RR = 2.08×**	RR = 1.65×
– Role labels (drivers, dashers, shoppers)	17.6%	31.1%	8.5%	RR = 1.76×	RR = 0.48×
– Null-subject constructions	1.19%	5.03%	1.30%	RR = 4.24×***	RR = 1.09× (n.s.)
First-person “I / me / my” per 1,000 w (contrast)	10.13	9.84	70.14	flat (d = −0.01)	d = +1.10*** (~7× rate)
***Worker petitions and supplications***					
Any company-directed petition	6.1%	17.5%	1.5%	RR = 2.87× (φ = 0.18)***	RR = 0.24×
– Financial petitions	3.9%	10.1%	0.09%	RR = 2.61×***	RR = 0.02×
– Algorithmic-financial petitions	1.8%	5.6%	0.0%	RR = 3.16×***	absent
– Pleas for humane treatment	1.2%	5.3%	0.0%	RR = 4.49×***	absent
Any supplication marker	3.1%	7.7%	0.65%	RR = 2.47× (φ = 0.10)**	RR = 0.21×
– “please”	1.04%	4.44%	0.65%	RR = 4.28×	RR = 0.62×
– “if you can”	0.15%	0.89%	0.0%	RR = 5.99×	absent
– “so we / drivers can continue working”	0.15%	0.59%	0.0%	RR = 3.99×	absent
Any ultimatum / exit-threat marker	0.59%	1.78%	0.2%	RR = 3.00× (small counts)	RR = 0.34×
***Compulsion and obsession (the Reddit signature)***					
Compulsion lexicon (addicted, hooked, can’t stop)	0.1%	0.9%	12.7%	RR = 9× (small counts)	RR = 127×***
Broader obsession / can’t-stop lexicon	0.7%	1.8%	14.1%	RR = 2.6× (n.s.)	RR = 19×***
– Self-naming as addicted	0.0%	0.0%	11.5%	absent in both	corpus-exclusive
***Register and stylistic markers***					
Mean review/post length (words)	38	75	36	1.97× (d = +0.48)***	d = −0.05 (n.s.)
Questions per 1,000 words	0.20	1.18	59.66	5.95× (d = +0.22)*	d = +0.20 (~300×)
Ellipses (…), % of texts containing ≥ 1	1.48%	5.03%	7.35%	RR = 3.39× (φ = 0.10)***	RR = 4.96×

### 4.1 Intense affective negativity and powerlessness

Sentiment, valence, rating, and LIWC Tone all moved sharply in Group B with large effects (d = −0.58 to −0.88); star ratings dropped by more than a full star (3.70 → 2.62). VADER compound dropped from +0.30 to +0.01 (d = −0.58); RoBERTa labeled 38% of Group B reviews negative vs 10% of Group A (RR = 3.67×) and only 17% positive vs 41% (RR = 0.42×); LIWC Tone fell from 76.1 to 52.5 (d = −0.70). NRC-VAD valence dropped (d = −0.50). Group B sentiment is uniformly negative. Reddit is ambivalent (VADER compound +0.23, between Group A and Group B), consistent with the peer-confessional register in which workers discuss both what attracts them and what harms them. NRC-VAD dominance (Reddit +0.04 vs Group A +0.09) and LIWC power (Reddit 0.79 vs Group A 3.64) collapse in the Reddit corpus – with the employer no longer in the audience, there is no one to blame for continued participation in what they perceive as harm.

### 4.2 Anger, disgust, fear, and sadness

NRC Intensity captured a roughly twofold elevation in Group B across the four negative-emotion families—anger (d = +0.40), fear (d = +0.52), sadness (d = +0.44), and disgust (d = +0.51). GoEmotions annoyance and disapproval rose roughly three-fold (RR = 2.86× and 3.33×); Hartmann’s RoBERTa-7 classifier assigned anger to 9.8% of Group B reviews vs 3.0% (RR = 3.31×). LIWC also showed a loss of positive affect: LIWC emo_pos and tone_pos halved (d = −0.40, d = −0.50). Similarly, Hartmann joy dropped from 30.6% to 15.7% (RR = 0.51×) and GoEmotions admiration from 16.0% to 4.1% (RR = 0.26×). Predictably, in the Reddit corpus, sadness (8.9%) matches Group B’s (8.0%).

### 4.3 Us-vs-them framing

Group B adopts the us-vs-them frame at substantially higher rates than Group A. On LIWC, the first-person plural pronouns we, us, our rise 3.8-fold (0.06% → 0.23% of words; d = +0.25, *p* < 0.01), and the third-person plural they, them, their roughly doubles (0.42% → 0.80%; d = +0.27, *p* < 0.01). Outgroup animosity and moral-emotional language reveal a perceived threat to one’s identity as an honorable person and a desire to feel superior in order to alleviate guilt (Rothschild and Keefer, 2017), and increase engagement (Rathje et al., 2021; Brady et al., 2017).

### 4.4 Increased preoccupation with the work

Group B writes reviews twice as long as Group A (d = +0.48) and produces nearly 3× as many very long reviews (14.6 vs 5.2%). Group B reviews are simultaneously longer, more cognitively elaborated, and more emotional, expressing an intense preoccupation with the work, characteristic of the betrayal bond.

### 4.5 Betrayal and abuse language and moral-foundation violation

On the eMFD, the foundations of fairness and authority each reversed from upheld in Group A to violated in Group B (d = −0.28 to −0.45, all *p* < 10^-4^). Care, already negatively valenced in Group A (−0.33), becomes substantially more negative in Group B (−1.12). Loyalty alone remains positively valenced in both groups (Group A = +1.70, Group B = +0.50) despite a significant decline (d = −0.41)—the foundation that would have to invert for the bond to break does not invert. Workers feel wronged but voluntarily remain bonded. The pattern replicates in the Reddit corpus: loyalty (+0.73) is diminished but remains positively valenced (d = −0.27 vs Group A); care is invoked as violated (−1.16), comparable to Group B’s −1.12. Workers who name themselves as addicted on Reddit and workers who indict the platform from Glassdoor both score above the zero of indifference on the loyalty foundation. The betrayal-frame lexicon—mistreatment vocabulary (abuse, punish, threat, cheat, victim)—appeared in 13.9% of Group B reviews vs. 4.3% of Group A (RR = 3.24, *p* < 0.001), with the punishment subcategory alone present in 7.4% of B reviews.

### 4.6 Confusion and denial: ellipses and avoiding the “I”

This intense preoccupation with the activity also manifests itself in confusion and denial. Confusion registers in elevated ellipses (RR = 3.39×, *p* < 0.001)—the trailing thought, the reluctance to admit the extent of absurdity. Denial registers in Group B’s avoiding using “I.” Even though their reviews are twice as long, they refer to themselves by using proxies—“you,” “people,” “drivers/dashers/shoppers,” or null-subject constructions (45.0% of Group B reviews versus 28.9% of Group A; *p* < 0.001, *φ* = 0.16). Null-subject openers (“Driving 12 hour days for $60”) rise 4.24× in Group B. First-person density itself is indistinguishable between groups (10.13 vs 9.84; d = −0.01, *p* = 0.86). On Reddit, the pattern reverses and “I” density rises seven-fold (70.14 per 1,000 words) while role labels fall to 0.76 per 1,000 words, and null-subject constructions to 1.3% of texts. With peers, workers acknowledge their painful experience and ask for advice.

### 4.7 Pleas and supplications to the platform

Company-directed petitions appeared in 17.5% of Group B reviews versus 6.1% of Group A (RR = 2.87, *p* < 0.001), with the largest growth in pleas for humane treatment (RR = 4.49×). Explicit supplication markers (please, if you can, kindly, help us, need to live) appeared in 7.7% of Group B vs. 3.1% of Group A (RR = 2.47, *p* = 0.001); “please” rose 4-fold, “if you can” rose 6-fold. Ultimatum and exit-threat markers (I quit, we are leaving, you’ll lose drivers) were essentially absent in both groups (under 2% in Group B). Group B negotiates from inside the bond, not from a position of credible exit.

### 4.8 Compulsive engagement, obsession, and the bodily-mental toll

The defining feature of the Reddit corpus is explicit mention of compulsion. The compulsion lexicon (addicted, hooked, cannot stop, sucked in) appeared in 12.7% of Reddit posts versus 0.9% Group B and 0.1% Group A. The addiction subcategory alone—workers self-diagnosing as addicted—accounts for 11.5% of Reddit posts (essentially zero in both review corpora) and is convergently corroborated by LIWC’s independent substances-and-dependency vocabulary (15–20 × elevation). The peer-confessional setting accommodates explicit naming of consequences the employer-review genre suppresses: LIWC health vocabulary appears in 21.5% of Reddit posts (RR ≈ 5 × vs. Group A); mental-health vocabulary in 14.6% (RR ≈ 146×). One in five Reddit posts mentions physical-health concerns; one in seven mentions mental-health concerns.

## 5 Discussion

### 5.1 Bonded participation: outrage and outgroup animosity increase engagement

The engagement of Group B workers is best described as bonded participation—they keep talking about the original promise that the job would make them feel independent, important, and valuable to the community. On the eMFD, the loyalty foundation that would have to reverse for the bond to break does not reverse, even as fairness and authority cross from positive valence in A into outright violation in B, and care intensifies further into the negative. Workers are disgusted: “WOW. How some of the things you guys do are even allowed is beyond me. How does a GROUP of people agree to do the things you guys do? It’s pretty unbelievable.” Outrage and disgust are visceral, high-arousal negative emotions we feel when our identity as a moral person is threatened (Rothschild and Keefer, 2017; Brady et al., 2017). Outgroup animosity is contagious and engaging, and we see workers use the adversarial framing of “us vs. them” more than twice as often (“we drivers bust our butts for you for not much pay,” “You guys are ABSOLUTELY THE WORST PAYING GIG DELIVERY EMPLOYER. We are out here suffering”). Feeling outraged helps us feel morally superior to our adversaries and increases our emotional investment and engagement (Rathje et al., 2021). Group B ask more rhetorical questions, exclaim, and shout in ALL CAPS: “Try driving for a day to actually see what it’s like and the loss of money, wear and tear you expect your drivers to take!” “NOTHING YOU CAN DO ABOUT ANY OF THIS EXCEPT FOR SUFFERING OR QUITTING!!!”

### 5.2 Sadness, supplications, and shame in the reviews

At the same time, feeling morally superior to the unfair employer does not last long because these workers know they are not leaving. We observe a pervasive feeling of sadness. The instruments showed that the Group B workers feel negative and powerless. In addition, they supplicate the employers who they know betrayed their promises; “Please pay your top tier shoppers more and give bonuses,” “Please pay your drivers more fairly,” “More money and more work please,” “Please consider offering a medical insurance to those putting in more than 30 h a week. Thank you!” “We are not machines! Thank you,” “I’m so sad about going into further debt delivering food to make a few bucks. Two cars ruined. No money for repairs. Please Uber Eats pay for my repairs.” Workers lower themselves in abjection: please, thank you, if you can are not politeness markers but attempts to engage the logic of reciprocity with a more powerful person or entity (Auyero, 2001). Workers preemptively express gratitude for favors not yet granted, hoping the management will respond by increasing pay.

Appeals for help follow the same logic. Instead of demanding fair compensation for their labor and the use of their own resources, workers ask the management to “help” them: “Gas prices are getting where I cannot afford to work anymore. Help pay for ½ of our gas so we can continue to work,” “Help with gas to top dashers with high acceptance rate no matter how far,” “Please help your delivery drivers and be kind to them,” “More gas price help, or increase base batch pay.” They implore the management to treat them humanely, as unique individuals, as they were promised: “you have to understand,” “you need to treat us better,” “listen to people and treat people with respect,” “treat workers better,” “actually care about your staff,” “listen to your people more,” “listen to shoppers,” “listen to your employees,” “more understanding.” By lowering themselves, workers literally illustrate Guy Standing’s argument that “the precariat consists of supplicants. The original Latin meaning of precarious [precarius] was ‘to obtain by prayer.’ That sums up what it is to be in the precariat: having to ask for favors, for help, for a break, for a discretionary judgment” (Standing, 2018). In this new iteration of precarious work, workers supplicate companies to “help” them by compensating them for their work and resources just so they can continue working for these same companies (rather than pay bills or buy food).

Workers are in a constant imaginary, one-sided dialogue with their employers, admonishing them and supplicating them, but also shielding themselves from the shame of continuing to engage with cognitive distortions. We see confusion and denial in these reviews, which are characteristic of the betrayal bond (Carnes, 1997, p. 194). Confusion registers in the many ellipses, which signal hesitation and reluctance to finish the thought in order to persist in this magnetic activity: “door dash pays garbage for a 60 km round trip for 10$… a cab would be 160.00 for this kind of trip,” “Low base pay while the company rakes in 2 billion a year…” Denial manifests itself in avoiding saying “I” and talking about the absurd and painful reality of losing money while working by using “you” or “people,” or “dashers,” “shoppers,” and “drivers.” The rise in null-subject constructions reflects the same need to deny one’s own participation in harm. Unlike Spanish or Italian, English requires that the subject of the action be named, but the intensely engaged worker drops the “I” in “Driving 12 h days for $60,” “Spending more on gas than you make,” “Lose money”—and continues doing these very things, against all logic.

### 5.3 The pleasure of feeling “productive”

That the promise of agentic, self-directed, and fun work resonates with so many people so strongly—6 million signed up for DoorDash in 2024 alone—is an indicator of how precarious work has become: uncertain, unstable, and insecure, with workers bearing the risks as opposed to companies or the state (Kalleberg, 2009, 2018). Approximately 44% of US workers age 18 to 64 are in precarious low-wage jobs (Ross and Bateman, 2019). In addition to the offshoring and outsourcing of manufacturing, tech, and customer service jobs, precariousness has expanded into academia, the school system, and even into previously protected jobs such as prep cooks, hospital nurses, and janitors (Gerstein, 2024). As historian Eric Baker notes in “Elon Musk is the world’s richest man. Why is he sleeping on an office floor?” (2025), having a job and appearing constantly busy “have become a status symbol: If the rich of the Gilded Age had conspicuous consumption, flaunting their freedom from toil, the rich of our new Gilded Age have conspicuous work”. Working all the time is the new luxury, a superpower, a mark of righteousness and moral superiority.

Indeed, working, or at least, being “productive” is a moral imperative in our society. Max Weber observed in *The Protestant Ethic and the Spirit of Capitalism*, “Waste of time is thus the first and in principle the deadliest of sins” (Weber, 1992, p. 104). One driver on Reddit repeats this almost verbatim: “it just feels like a sin to be doing nothing.” Many drivers thank the apps for saving them from the sin of laziness and recall their fathers and grandfathers who told them to never refuse work. If it were not for the apps, they would stay home and play videogames: “Better than sit around the house and play videogames lol… DoorDash can fill the otherwise unimpressive hours of my day, when I would just be lazying about.” Watching YouTube and listening to a podcast is felt as laziness: “I’ll be at home watching YouTube videos or playing the game anyways. Why not drive and listen to my YouTube,” “No idea why it’s so fun at times but it’s nice just driving around while still feeling productive.” Even sleeping is laziness: “If I wasn’t out hustling, I’d most likely be at home sleeping or watching TV. Being lazy is easy and this keeps me moving.” The apps allow one to procrastinate and not feel guilty about it: “I’m a student and I procrastinate on my projects and homework by doing batches. I make it into a game, to beat the estimated time, to try to keep my 5.0, etc. I’m glad I found a better alternative to procrastinating with video games or binging shows!” Thus, the apps turn “lazy” time of relaxing at home into “productive” time, even though this “productivity” benefits the company rather than the worker.

In return, drivers receive the validation they can check at any moment—the always-growing personal “stats”: deliveries made, on-time deliveries, points and statuses earned. They proudly share their stats on forums as proof of self-discipline and work ethic. Personal quantification increases engagement and output, and companies use this to their advantage (Etkin, 2016). By design, this validation always accrues, including the earning, even though half of the amount the app displays is actually expenses or losses (Big Lake Data, 2024). As Baudrillard noted, in today’s growth-oriented societies, even losses, when properly recorded, can be perceived as gains (Baudrillard, 1998, p. 41). When drivers realize that these metrics are irrelevant outside the apps and their online community, their pleasure at feeling “productive” dissipates.

### 5.4 Pain and pleasure: the laughing-crying emoji

Workers reach for gaming and gambling metaphors to describe their time on the app. Deliveries are like missions and quests of videogames such as Grand Theft Auto, Crazy Taxi, and Pokémon Go, and explain, “You got the bells and notifications. Percentages. All that video game shit to keep you in it.” Other drivers compare the apps to slot machines in casinos: “the app is designed to be like a casino,” “It’s very much like the slots in Vegas,” “the constant refreshing is like pulling the lever at the casino.” It is telling that users compare delivery apps to slots because new slots with virtual reels are engineered to stretch the players’ money for sessions that can last for days, and utilize the illusions of constant near-misses and constant “winning”: for each amount wagered, players “win” 80% of it back. Today, slots generate the gambling industry’s profits, and account for 90% of gambling addicts in rehab facilities (Schüll, 2012: 5). App drivers explain the intermittent reward mechanism to each other: “For noobs the algorithm is designed to spike your dopamine and create an addiction,” “apps and companies actually pay big money to casino/gambling/slot machine researchers and try to mimic their design choices, notification sounds, and incentives/rewards/feedback interfaces to make them addictive.” Game designers have argued that games give ordinary people the opportunity to experience “flow,” an active state of skilled mastery, without the hard work or special skills it requires (Jones et al., 2014). But while flow is “escape forward”, machine zone or “dark flow” is a passive dissociative state of self-forgetting or “escape backwards” (Csikszentmihalyi, 1990).

The laughing-crying face, the most frequent emoji app workers use, tells us that like other addictive products, apps bring both pain and pleasure. While smokers or social media users report enjoying using these products, they also wish they had never been invented, unlike nonaddicting products such as hairbrushes (Haidt, 2024). Drivers describe their feelings for the apps as love - hate: “my husband thinks I’m addicted to instacart and I hate to admit it but I am a little addicted… making the money, shopping, getting a good batch, checking my stats, loving that dopamine 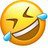.”^1^ It would seem like a fair exchange of labor, time, and resources for the pleasure that the app provides, but this is not the case. Neuroscientists explain that exposure to addictive products shifts the brain’s homeostatic balance between pleasure and pain to the side of pain. Usual activities become boring and unsatisfying, so users must return to the addictive activity for relief (Lembke, 2021; Grisel, 2019).

In other words, drivers give their time, resources and labor to the app, but they also feel worse than before they started on it. This user makes clear that intense pleasure turns to an even more intense pain: “The excitement I feel in my brain when I get a good batch is too much, it’s an influx of 5 days of dopamine all at once mixed with coke…but the lows oh man the lows…” Indeed, users confess being obsessed with the apps, saying, for example, “I can’t stop thinking about it at all times,” and “It’s all I want to do!!” Everything else becomes an obstacle to being on the app, for example, actual job: “I’ve gotten to the point where I feel like my full time job is getting in the way of my door dashing”; general life obligations and chores: “I have started to use door dash as a reward. If I get the shit on my to do list done, only then I get to do doordash,” and even loved ones: “I literally hate to respond to my bf’s texts for fear of missing a good batch,” “to get away from my wife,” and “to take a break from my kids, everyone is on their devices anyway.

### 5.5 Therapy and escape of the machine rhythm

Time on the app feels like hypnotic and numbing experience (Schüll, 2012; Dixon et al., 2017). The machine sets the rhythm and the person follows it. Leslie Hook, who studies the apps and works for them says that “In that state, [drivers] are literally just listening to the sounds. Stopping when they said stop, pick up when they say pick up, turn when they say turn. You get into a rhythm of that, and you begin to feel almost like an android” (Hook, 2017). One user feels as a mindless as a clam: “There are days when I am just happy as a clam driving around no matter what kind of orders I get.” The clam image illustrates the trance-like absorption afforded by the apps. Following the app’s directions is “actually relaxing,” “real soothing,” “mindless,” say the users. They forget about the time and self: “it’s my escape,” “It’s my therapy and keeps my mind busy,” “It relieves my anxiety,” “I too struggle with anxiety and it gives me purpose.” Instead of the promised freedom, one is simply following the machine.

### 5.6 The sadness and shame of feeling powerless to stop

The crucial difference between app drivers and gamblers is that gamblers do not feel betrayed. Gamblers know that they come to the machines to experience the loss of their money and time as the only thing they control, unlike the losses in real life, which cannot be controlled at all. Winning or losing does not matter as long as the machine provides the absorptive escape, the numbness that allows them to forget their problems. As one gambler put it, “At the machines, I do know: either I’m going to win, or I’m going to lose. I do not care if it takes coins or pays coins: the contract is that when I put a new coin in, get five new cards, and press those buttons, *I am allowed to continue*. If you cannot rely on the machine, then you might as well be in a human world where you have no predictability either” (Schüll, 2012: 12, our italics).

App drivers are just as hooked, and just as sad, as their supplications in the reviews already showed. Just like gamblers, all they want is to *continue*: “We do not even break even on gas. That’s sad. Please do better so I can *continue* to work I have a kid to feed and we are almost next to homeless. This is not fair”; “Pay more to your employees so they can *continue* working for you”; “Help pay for ½ of our gas so we can *continue* to work.” App users become as sad and fatalistic as gamblers, because being unable to stop losing money causes real problems, shame, and the need to hide use, leading to isolation. In the reviews this shame registers in avoiding saying “I.” On Reddit, users confess their self-imposed isolation from family and friends to hide their app use and reach for the language of drug addiction: “I’m constantly planning on how to get my fix and keep my hustle from others. I go through great lengths to hide my dashing use from family and friends. I start panicking toward the end of the dash if it does not let me extend my current dash. I think I need DD meetings,” “I have a family so I feel kind of bad for going out because I already work full time but I feel bored at home when I do not go out,” “My family said they barely see me anymore these last two weeks,” “I barely eat or sleep. I have not spoken to my friends or family in over a year.”

Gamers and gamblers were never promised freedom, autonomy, or income, so they treat the consequences of their use as a failure of self-discipline and blame themselves. App drivers do a lot of real-life, hard, and risky work, and use their own resources hoping that the promise of dignified work was real. When the promise fails to come true no matter how hard they worked for it, workers reach for the language of betrayed intimacy, abuse, manipulation, and cheating. They intuit that instead of the promised freedom and autonomy, they received, and stayed for, the opposite—a non-deciding, non-strategic, automatic mode. Bonded participation is the new frontier of surveillance capitalism. In its early days, this “loss of self-awareness, automatic behavior, and a total rhythmic absorption carried along on a wave of compulsion” (Zuboff, 2019, p. 281) resulted in the production and consumption of online content for the platforms. Now it can become real-life labor.

## 6 Theoretical implications

Emotional and affective surveillance technology can be used to monitor workers’ “unproductive” unhappy emotions and threaten their career advancement (Mantello and Ho, 2023). In the gig delivery industry, where labor is increasingly deskilled and career advancement nonexistent, this technology appears to elicit these previously undesirable, throwaway emotions of sadness and outrage and convert them into continued participation in tightly scheduled and surveilled, uncreative, and risky work. When tech companies started collecting their users’ personal data, they spoke of it as “digital exhaust” and “digital breadcrumbs”—something no one could possibly ever need or think that collecting it could be “an act of exploitation, expropriation, or plunder” (Zuboff, 2019, p. 90). Affective surplus can too be collected and put to use. Policy that addresses algorithmically controlled gig labor solely through wage and classification reform will leave this mechanism intact. Our analysis supports proposals (Dubal, 2023; Teachout, 2023; Human Rights Watch, 2025) to ban algorithmic wage assignment. This measure will disrupt the intermittent reward mechanism which keeps drivers working in spite of perceived harm to themselves.

## 7 Limitations

Several limitations qualify these findings. The two corpora are non-random samples assembled from public web sources. Glassdoor reviews are written by workers who chose to write a review. The Reddit corpus was assembled around discussions of app use against self-interest and may therefore overrepresent compulsion-related discourse. Cross-corpus comparisons are interpreted descriptively rather than as controlled contrasts. Computational sentiment and emotion instruments cannot directly read intent or pragmatic meaning, and our study-specific lexicons (betrayal and compulsion vocabulary, self-shielding strategies, supplications) require interpretive judgment in their construction. Although we audited each lexicon on random samples and the inter-rater reconciliation on the manual coding was very high, residual interpretive ambiguity is inherent in the method. The text data analyzed here capture workers’ unprompted self-narration, but they are static and retrospective; a natural next step would be in-depth interviews with delivery workers that use the constructs identified in this study to probe more deeply into how the relationship between platform design, emotion, and continued participation develops over time within individual workers. Within these limitations, the convergence of the findings across two corpora, three platforms, eight validated instruments, and four study-specific analyses supports our argument that the intense affective engagement of the sizable minority of workers who do most of the labor for gamified pay-to-work food delivery platforms can best be described as bonded participation.

## 8 Conclusion

The angry-and-sad-but-staying minority of gig delivery workers documented in this study—roughly one-third of current-employee reviews on Glassdoor, and the population most visible in peer-confessional Reddit communities—exhibits an affective signature that no single existing framework of gig-work motivation, engagement, or consent fully captures. These workers name the work as harmful and continue to work. They retain measurable loyalty on a validated moral-foundations instrument while fairness and authority cross from positive valence into outright violation, and care intensifies further into the negative. They avoid using “I” to shield themselves from the absurd reality of working hard and not making or losing money, supplicate the employer to help them continue working, and self-diagnose as addicted. The concept of betrayal bond (Carnes, 1997) and behavioral surplus (Zuboff, 2019) provide the analytic apparatus that holds these findings together—not as a diagnostic claim about individual workers but as a structural description of a relational pattern in which the job design elicits and sustains continued participation under conditions the workers themselves name as harmful. In a gamified gig labor model that has eliminated career advancement and standard employment protections, workers’ outrage, sadness, and shame are the affective surplus turned into continuous productivity.

## Data Availability

The original contributions presented in the study are included in the article/Supplementary material, further inquiries can be directed to the corresponding author.

## Footnotes

1 All emojis designed by OpenMoji – the open-source emoji and icon project. License: CC BY-SA 4.0.
