# Supplementary material for: Angry and “productive”: engagement in gamified precarious work
Source: Front Psychol. 2026 Aug 7;17:1833833. doi: 10.3389/fpsyg.2026.1833833 (PMC13495201; doi:10.3389/fpsyg.2026.1833833)
Supplement: Supplementary file 1 [file Data_Sheet_1.docx]

**Contents**

Appendix A. Coding manual for Group B classification

Appendix B. Distancing pronouns: operational rules and audit

Appendix C. Petitions and supplications: operational rules

Appendix D. Compulsion and obsession lexicon

Supplementary Table S1. Validated computational instruments — full statistics

Supplementary Table S2. Study-specific linguistic analyses — full statistics

**Appendix A. Coding manual for Group B classification**

Group B (intense and conflicted engagement) was identified from the eligible pool of current-employee reviews (n = 982) by a single inclusion criterion: the worker remarks that the costs of participating — gas, vehicle wear, and related expenses — meet or exceed the pay, or that pay is too low to cover them, and does so with discernible emphasis or emotion (evaluative or affective wording, intensifiers, capitalization, exclamations, rhetorical questions, repetition). Two raters read every current-employee review and coded it independently, meeting at regular intervals to align their application of the criteria. At final reconciliation they coincided on 97% of reviews (Cohen's κ ≈ 0.93; Landis and Koch, 1977: "almost perfect"). The remainder was resolved by discussion to consensus.

**A.1 Inclusion criteria (Group B)**

A review is coded as Group B if all three conditions are met:

(i) The worker is a current employee (not a former/departed worker).

(ii) The worker explicitly states that the costs of doing the work meet or exceed the pay, or that the pay does not cover gas, vehicle wear, time, or expenses.

(iii) The statement is made with affective or evaluative emphasis: intensifiers (so, very, extremely, absolutely), capitalization, exclamations, rhetorical questions, repetition, or evaluative wording (terrible, awful, abusive, scam, etc.).

**A.2 Exclusion criteria**

A review is excluded from Group B if any of the following apply:

(i) The reviewer is marked as a former employee.

(ii) The cost-exceeds-pay relationship is not raised.

(iii) Losses are framed as occasional or hedged with qualifiers such as "sometimes," "can be," "depending on the area," etc.

(iv) Complaints focus on customers, app malfunction, route problems, or other issues without the cost-exceeds-pay framing.

**A.3 Anchor examples — Group B (included)**

Each example below was coded as Group B by both raters at the first pass.

*Pay barely covers gas. Drive 12 hours and walk away with $40 after expenses. This is a scam.*

*I LOSE MONEY EVERY SHIFT. Gas is $4, the orders pay $3. Math doesn't lie. Why are we still here??*

*It used to be okay but base pay is now $2 for 8 miles. After expenses I make less than minimum wage. Please pay your drivers.*

*They expect us to deliver for $2.25 base pay. I can't even buy a single gallon of gas for that. We are not robots, we have bills.*

*I work 60 hours a week and after taxes, gas, oil changes, and brake jobs, I'm making $5 an hour. This is not sustainable. Treat us like humans.*

**A.4 Anchor examples — not Group B (excluded)**

Each example below was excluded from Group B at the first pass. The exclusion rationale is noted after each.

*Pay is fine. Some days are slow but overall I make good money on weekends. (no cost-exceeds-pay framing; positive overall)*

*Customers can be rude sometimes. Other than that the work is whatever you make of it. (complaint focused on customers; no pay/cost framing)*

*Pay isn't great but the flexibility makes up for it. (hedged; no affective emphasis; flexibility valued)*

*Sometimes you make $5 an hour, sometimes you make $25. Depends on the market. (hedged with "sometimes"; framed as variable)*

**A.5 Funnel by platform**

After eligibility filtering and Group A/B coding, the platform breakdown is: DoorDash (Group A n = 495, Group B n = 235, B = 32.2% of current-employee), Instacart (Group A n = 71, Group B n = 53, B = 42.7% of current), Uber Eats (Group A n = 78, Group B n = 50, B = 39.1%). Across the three platforms, n(Group A) = 644 and n(Group B) = 338, with Group B comprising 34.4% of current-employee reviews. See Table 1 in the main text for the full funnel.

**Appendix B. Distancing pronouns: operational rules and audit**

The four distancing markers operationalize linguistic shielding documented in the distanced self-talk literature (Orvell et al., 2017; Orvell, Ayduk, Kim, Gelman, and Kross, 2019), by which speakers in betrayal bonds use distancing proxies in place of first-person "I" to rationalize continued participation in harm without claiming the experience as their own. Each marker was computed at per-1,000-word density and per-text prevalence; a combined cluster index aggregates any marker hit.

**B.1 Operational rules**

**Marker 1 — Generic "you" preceded by a third-person platform subject.** Regex: \b(they|the app|the apps|DoorDash|Instacart|Uber|UberEats|Lyft|management|customers?|the algorithm)\b[^.!?]{0,50}\byou\b. Captures sentences where the platform or its actors are the explicit subject and "you" follows within 50 characters as the worker stand-in.

**Marker 2 — Generic "people" as proxy for the worker.** Regex: \bpeople\b (in worker-self-reference contexts). False-positive filter excludes references to customers, friends, or family.

**Marker 3 — Generic role labels.** Regex: \b(drivers?|dashers?|shoppers?|couriers?)\b. Counts the worker referring to themselves as a member of the role class rather than as "I."

**Marker 4 — Null-subject constructions.** Regex matching subjectless gerund or imperative-form clauses describing the worker's own labor or expense at sentence boundaries: ^(driving|dashing|delivering|working|spending|losing|earning|making|paying|wasting|hauling)\b. Captures phrases like "Driving 12 hours for $60," "Spending more on gas than you make," "Lose money."

**B.2 Audit on random samples**

Each marker was audited on a random sample of 50 hits per corpus. Precision (proportion of hits judged true positives by manual inspection):

| **Marker** | **Group A precision** | **Group B precision** | **Reddit precision** |
| --- | --- | --- | --- |
| Generic "you" (after third-person) | 92% | 94% | 88% |
| Generic "people" | 86% | 88% | 82% |
| Role labels | 90% | 92% | 90% |
| Null-subject constructions | 94% | 96% | 92% |

First-person singular density ("I / me / my" per 1,000 words) was measured as a validating contrast. If distancing is the operative mechanism, the cluster should rise in Group B while first-person singular remains flat — which is what the data show (A: 9.11; B: 9.85; d = +0.03, p = .73). The cluster rise in Group B (RR = 1.56× vs A) cannot be explained by reduced "I" use.

**Appendix C. Petitions and supplications: operational rules**

Company-directed petitions are sentences in which the worker demands, requests, or begs action of the platform. We identified petitions using four syntactic constructions and three semantic categories.

**C.1 Syntactic constructions**

(i) Direct imperative addressed to the platform: "Pay drivers more," "Fix the app."

(ii) Soft directive prefixed by "please": "Please fix the app," "Please pay your drivers."

(iii) Modal demand with third-person platform subject and deontic modal + action verb: "They should treat drivers fairly," "DoorDash needs to raise the base pay."

(iv) Embedded passive: "Drivers should be paid more," "Acceptance rates should be abolished."

**C.2 Semantic categories**

Each petition was classified by action verb into one of three categories:

Financial: pay, compensate, reimburse, raise, increase, return, restore.

Algorithmic / technical: fix, update, stop, change, improve, abolish (the algorithm/tier/acceptance rate).

Humanization: treat, value, respect, listen, acknowledge, recognize, care, prioritize.

**C.3 Supplication markers**

A separate lexicon captured explicit supplication: "please," "if you can," "kindly," "help us / drivers," "so we / drivers can continue working," "need + survival" ("to live," "to make it"), "barely making it," "next to homeless." This category captures the abjection register identified in the Discussion (Auyero, 2001; Standing, 2018).

**C.4 Ultimatum and exit-threat markers (validating contrast)**

A contrasting lexicon captured exit-threats: "I quit," "we're leaving / done," "you'll lose drivers," "we won't accept," "going elsewhere," "we deserve / have a right." This category operationalizes Carnes's (1997) account of bonded negotiation as the contrast term: workers in betrayal bonds petition for tolerable conditions from inside the bond rather than threatening to exit it. Group B's supplication-to-ultimatum ratio is ~4:1; Group A's is ~5:1; on Reddit, both registers are near-absent (peer-confessional rather than employer-directed).

**Appendix D. Compulsion and obsession lexicon**

Motivated by Carnes's (1997) identification of obsession and preoccupation as core sustaining features of the betrayal bond, the lexicon comprises five subcategories. Words were matched with word-boundary rules; negative-context filters excluded references to other people's addictions ("my brother is addicted") or non-app contexts.

**D.1 Subcategory word lists**

**Addiction and dependency:** addicted, addiction, addict, hooked, fix, withdrawal, dopamine, dopamine hit, binge, junkie, crack, heroin, cocaine, dealer.

**Can't-stop / can't-quit constructions:** can't stop, cannot stop, couldn't stop, can't quit, can't leave, can't get out, no choice but to, only option, last resort, trapped, golden handcuffs, sucked in, sucks me in, reels me back, keep coming back, kept coming back.

**Cognitive obsession:** obsessed, preoccupied, consumed, fixated, haunts, all I think about, in my head, can't stop thinking, mind is on, always on my mind.

**Temporal saturation:** every single day, all day every day, around the clock, 24/7, non-stop, day and night, 80-hour week, working constantly, never stop, every waking hour.

**Sucked-in / trapped:** sucks me in, sucked in, can't escape, no escape, dead end, stuck, stuck doing, stuck with, stuck in.

**D.2 Reddit-exclusive subcategory**

Self-naming as addicted (e.g., "I'm addicted to DoorDash," "I think I need DD meetings," "I hit the markers for addiction") appears in 11.5% of Reddit posts and is essentially absent in both review corpora (0% in A and B). This is reported as the cleanest single piece of evidence for compulsion self-recognition on Reddit and the genre-specific signature of the peer-confessional register.

**D.3 Convergent LIWC substances signal**

LIWC-22's substances category, an independent dictionary, was 15–20× more elevated on Reddit than on either review corpus (Reddit M = 0.088, vs A = 0.004, B = 0.006). The convergence of the study-specific lexicon and the LIWC validated category confirms that the compulsion signature is not an artifact of the study-specific word choices.

**Supplementary Table S1. Validated computational instruments — full statistics**

Welch's two-sample t-tests for continuous measures; chi-square tests for categorical measures (% of texts above threshold). Effect sizes: Cohen's d with 95% CIs (continuous), risk ratios with 95% CIs (categorical). p-values Benjamini–Hochberg FDR-corrected across all comparisons in Tables 3, 4, S1, and S2.

| **Measure** | **Group A** | **Group B** | **Reddit** | **B vs A** | **R vs A** |
| --- | --- | --- | --- | --- | --- |
| ***Sentiment and overall polarity*** | | | | | |
| VADER compound (M) | +0.30 (0.45) | +0.01 (0.57) | +0.23 (0.55) | t = 8.07, d = −0.58 [−0.72, −0.45], p < .0001 | t = 12.4, d = −0.88 [−0.98, −0.78], p < .0001 |
| LIWC Tone (M) | 76.1 (28.0) | 52.5 (33.5) | 54.8 (39.4) | t = 10.8, d = −0.70 [−0.83, −0.56], p < .0001 | t = 13.4, d = −0.70 [−0.80, −0.60], p < .0001 |
| Strongly negative texts (compound ≤ −0.50), % | 6.2% (40/644) | 24.0% (81/338) | 9.4% (101/1076) | χ² = 53.4, RR = 3.86 [2.71, 5.49], p < .0001 | χ² = 4.50, RR = 1.51 [1.04, 2.20], p < .05 |
| ***Affective dimensions: NRC-VAD*** | | | | | |
| Valence | +0.18 (0.09) | +0.14 (0.10) | +0.12 (0.13) | t = 8.07, d = −0.50 [−0.63, −0.36], p < .0001 | t = 11.4, d = −0.60 [−0.71, −0.50], p < .0001 |
| Dominance (n.s. after symmetric cleaning) | +0.09 (0.07) | +0.09 (0.07) | +0.04 (0.10) | t = 1.52, d = −0.10 [−0.24, +0.03], p = .13 (n.s.) | t ≈ 9, d ≈ −0.54 (approx., post-cleaning), p < .0001 |
| Arousal (flat) | −0.07 (0.07) | −0.06 (0.07) | −0.05 (0.10) | t = 1.47, d = +0.09 [−0.04, +0.22], p = .14 (n.s.) | t = 0.10, d ≈ 0, p = .92 |
| ***Psycholinguistic structure (LIWC-22)*** | | | | | |
| Authentic (n.s. after symmetric cleaning) | 43.1 (29.4) | 41.0 (29.0) | 72.2 (32.2) | t = 0.95, d = −0.06 [−0.20, +0.07], p = .34 (n.s.) | t = 25.8, d = +1.27 [+1.16, +1.38], p < .0001 |
| Power (n.s. between A and B after cleaning) | 3.64 (3.62) | 3.79 (3.39) | 0.79 (2.33) | t = 0.63, d = +0.04 [−0.09, +0.18], p = .53 (n.s.) | t ≈ 20, d = −0.96 [−1.07, −0.86], p < .0001 |
| Cognitive processing (cogproc; n.s. after symmetric cleaning) | 10.22 (4.74) | 10.28 (4.62) | 10.69 (5.55) | t = 0.14, d = +0.009 [−0.12, +0.14], p = .89 (n.s.) | t = 1.62, d = +0.09 [−0.01, +0.19], p = .11 |
| Differ (contrastive) | 2.88 (2.43) | 3.39 (2.75) | 3.63 (3.45) | t = 2.33, d = +0.15 [+0.02, +0.28], p = .02 | t = 4.51, d = +0.21 [+0.11, +0.31], p < .0001 |
| Negate | 2.32 (1.96) | 2.97 (2.25) | 2.19 (2.46) | t = 3.24, d = +0.22 [+0.09, +0.35], p = .001 | t = 0.84, d = −0.04 [−0.14, +0.06], p = .40 |
| Certitude | 0.31 (0.84) | 0.50 (1.02) | 0.96 (1.66) | t = 2.39, d = +0.16 [+0.03, +0.29], p = .02 | t = 10.7, d = +0.32 [+0.22, +0.42], p < .0001 |
| emo_anger | 0.11 (0.45) | 0.07 (0.38) | 0.19 (0.74) | t = 0.90, d = −0.06 [−0.20, +0.07], p = .31 (n.s.) | t = 4.15, d = +0.13 [+0.04, +0.23], p < .001 |
| Pronoun "we" | 0.06 (0.40) | 0.23 (0.97) | 0.31 (1.77) | t = 3.05, d = +0.25 [+0.12, +0.38], p = .002 | t = 3.75, d = +0.17 [+0.07, +0.27], p < .001 |
| Pronoun "they" | 0.42 (1.10) | 0.80 (1.51) | 0.38 (1.77) | t = 3.78, d = +0.27 [+0.14, +0.40], p < .001 | t = 0.62, d = −0.03 [−0.13, +0.07], p = .54 (n.s.) |
| ***Discrete emotion (% of texts)*** | | | | | |
| Hartmann-RoBERTa-7 sadness | 4.5% (29/644) | 8.0% (27/338) | 8.9% (96/1076) | χ² = 4.94, RR = 1.77 [1.07, 2.93], p < .05 | χ² = 8.40, RR = 1.98 [1.30, 3.00], p < .01 |
| Hartmann-RoBERTa-7 anger | 3.0% (19/644) | 9.8% (33/338) | 6.5% (70/1076) | χ² = 20.5, RR = 3.31 [1.92, 5.71], p < .0001 | χ² = 8.39, RR = 2.10 [1.30, 3.39], p < .01 |
| Hartmann-RoBERTa-7 disgust | 2.0% (13/644) | 5.0% (17/338) | 6.8% (73/1076) | χ² = 6.40, RR = 2.49 [1.22, 5.10], p < .01 | χ² = 14.8, RR = 2.87 [1.68, 4.91], p < .001 |
| GoEmotions disappointment (n.s. after symmetric cleaning) | 1.2% (8/644) | 1.5% (5/338) | 4.4% (47/1076) | χ² = 0.10, RR = 1.19 [0.39, 3.62], p = .76 (n.s.) | χ² = 11.7, RR = 3.55 [1.68, 7.49], p < .001 |
| GoEmotions annoyance | 1.9% (12/644) | 5.3% (18/338) | 4.0% (43/1076) | χ² = 8.96, RR = 2.86 [1.39, 5.86], p < .01 | χ² = 5.51, RR = 2.13 [1.13, 4.00], p < .05 |
| Hartmann-RoBERTa-7 fear | 1.9% (12/644) | 3.8% (13/338) | 3.6% (39/1076) | χ² = 3.40, RR = 2.06 [0.95, 4.46], p = .06 | χ² = 3.62, RR = 1.94 [1.03, 3.66], p < .05 |
| Hartmann joy | 30.6% (197/644) | 15.7% (53/338) | — | χ² = 27.4, RR = 0.51 [0.39, 0.68], p < .0001 | — |
| GoEmotions admiration | 16.0% (103/644) | 4.1% (14/338) | — | χ² = 27.4, RR = 0.26 [0.15, 0.45], p < .0001 | — |
| ***NRC Intensity Lexicon (per-word intensity, mean per text)*** | | | | | |
| NRC anger intensity | 0.09 (0.15) | 0.15 (0.16) | 0.14 (0.18) | t = 6.04, d = +0.40 [+0.27, +0.53], p < .0001 | t = 6.92, d = +0.29 [+0.19, +0.39], p < .0001 |
| NRC fear intensity | 0.09 (0.15) | 0.19 (0.18) | 0.13 (0.18) | t = 7.83, d = +0.52 [+0.39, +0.65], p < .0001 | t = 4.95, d = +0.21 [+0.11, +0.31], p < .0001 |
| NRC sadness intensity | 0.12 (0.16) | 0.22 (0.18) | 0.14 (0.20) | t = 6.59, d = +0.44 [+0.31, +0.57], p < .0001 | t = 2.30, d = +0.11 [+0.01, +0.21], p < .05 |
| NRC disgust intensity | 0.06 (0.13) | 0.16 (0.18) | 0.11 (0.17) | t = 7.73, d = +0.51 [+0.38, +0.64], p < .0001 | t = 5.43, d = +0.32 [+0.22, +0.42], p < .0001 |
| ***Moral foundations (eMFD; sentiment)*** | | | | | |
| Care | −0.33 (0.45) | −1.12 (1.03) | −1.16 (0.92) | t = 13.5, d = −0.28 [−0.42, −0.15], p < .0001 | t = 17.5, d = −0.22 [−0.32, −0.13], p < .0001 |
| Fairness | +0.52 (0.60) | −0.51 (0.88) | +0.24 (0.78) | t = 18.7, d = −0.41 [−0.54, −0.27], p < .0001 | t = 7.91, d = −0.08 [−0.18, +0.02], p = .12 |
| Authority | +0.34 (0.62) | −0.91 (0.85) | +0.13 (0.80) | t = 20.8, d = −0.45 [−0.58, −0.31], p < .0001 | t = 4.94, d = −0.05 [−0.15, +0.05], p = .31 |
| Loyalty (the bond signature) | +1.70 (1.10) | +0.50 (1.16) | +0.73 (0.85) | t = 13.7, d = −0.41 [−0.54, −0.28], p < .0001 | t = 17.5, d = −0.27 [−0.36, −0.17], p < .0001 |
| ***Bodily and mental toll (% of texts)*** | | | | | |
| LIWC health | 4.3% (28/644) | 6.5% (22/338) | 21.5% (231/1076) | χ² = 2.07, RR = 1.51 [0.88, 2.59], p = .15 (n.s.) | χ² = 102, RR = 5.00 [3.41, 7.32], p < .0001 |
| LIWC mental health | 0.1% (1/644) | 0.3% (1/338) | 14.6% (157/1076) | χ² = 0.06, RR ≈ 2.0 (small counts), p = .81 | χ² = 89.0, RR ≈ 99, p < .0001 |
| LIWC Physical (M, per 1,000 w) | 0.69 (1.38) | 0.79 (1.37) | 1.87 (2.05) | t = 1.14, d = +0.07 [−0.06, +0.20], p = .26 (n.s.) | t = 13.2, d = +0.37 [+0.27, +0.47], p < .0001 |
| ***Engagement and excitement emotions (% of texts)*** | | | | | |
| GoEmotions excitement | 0.0% (0/644) | 0.0% (0/338) | 0.7% (8/1076) | absent in both | corpus-exclusive to R |
| Hartmann-RoBERTa-7 surprise | 0.5% (3/644) | 0.0% (0/338) | 10.7% (115/1076) | χ² = 1.58, n.s. | χ² = 71.6, RR ≈ 21, p < .0001 |
| GoEmotions amusement | 0.0% (0/644) | 1.2% (4/338) | 9.7% (104/1076) | small counts | corpus-exclusive to R |

***Note.*** *Continuous measures reported as M (SD); categorical measures as % (n / N). t and χ² are Welch and Yates-corrected respectively. All p-values shown are Benjamini–Hochberg FDR-corrected; q-threshold = .05. Cohen's d and risk ratios reported with 95% CIs in brackets. Cross-corpus comparisons involving Reddit are interpreted descriptively rather than as controlled contrasts.*

**Supplementary Table S2. Study-specific linguistic analyses — full statistics**

Chi-square tests of independence (Yates-corrected for cell counts < 5). Risk ratios with 95% CIs. p-values Benjamini–Hochberg FDR-corrected across all comparisons in Tables 3, 4, S1, and S2.

| **Measure** | **Group A** | **Group B** | **Reddit** | **B vs A** | **R vs A** |
| --- | --- | --- | --- | --- | --- |
| ***Betrayal-frame language*** | | | | | |
| Any betrayal-frame term, % of texts | 1.5% (10/644) | 5.7% (19/338) | 0.37% (4/1076) | χ² = 16.7, RR = 3.80 [1.78, 8.10], p < .001 | χ² = 7.05, RR = 0.25 [0.08, 0.79], p < .01 |
| Mistreatment vocabulary, combined | 4.3% (29/644) | 13.9% (47/338) | 0.74% (8/1076) | χ² = 30.0, RR = 3.24 [2.07, 5.07], p < .0001 | χ² = 26.6, RR = 0.17 [0.08, 0.38], p < .0001 |
| – Abuse / mistreat | 0.44% (3/644) | 2.37% (8/338) | 0.09% (1/1076) | χ² = 7.59, RR = 5.33 [1.43, 19.9], p < .01 | χ² = 1.45, RR = 0.21 [0.02, 2.01], n.s. |
| – Punish / penalize | 1.78% (12/644) | 7.40% (25/338) | 0.0% (0/1076) | χ² = 21.6, RR = 4.16 [2.11, 8.21], p < .0001 | absent on Reddit |
| – Cheat / scam / rob | 1.48% (10/644) | 4.73% (16/338) | 0.28% (3/1076) | χ² = 10.4, RR = 3.20 [1.45, 7.04], p < .01 | χ² = 5.51, RR = 0.19 [0.05, 0.69], p < .05 |
| – Threat / coerce | 0.30% (2/644) | 1.48% (5/338) | 0.09% (1/1076) | χ² = 4.62, RR = 4.99 [0.97, 25.7], p < .05 | χ² = 0.65, RR = 0.31 [0.03, 3.46], n.s. |
| ***Distancing pronouns and first-person voice*** | | | | | |
| Combined distancing cluster, % of texts | 28.9% (195/644) | 45.0% (152/338) | 16.3% (175/1076) | χ² = 25.9, RR = 1.56 [1.31, 1.85], p < .0001, φ = 0.16 | χ² = 40.8, RR = 0.56 [0.47, 0.67], p < .0001 |
| – Generic "you" | 15.3% (103/644) | 26.9% (91/338) | 3.0% (32/1076) | χ² = 19.7, RR = 1.76 [1.36, 2.27], p < .0001 | χ² = 80.3, RR = 0.20 [0.13, 0.29], p < .0001 |
| – Generic "people" | 3.7% (25/644) | 7.7% (26/338) | 6.1% (66/1076) | χ² = 7.62, RR = 2.08 [1.21, 3.57], p < .01 | χ² = 4.92, RR = 1.65 [1.05, 2.59], p < .05 |
| – Role labels | 17.6% (119/644) | 31.1% (105/338) | 8.5% (91/1076) | χ² = 24.0, RR = 1.76 [1.41, 2.21], p < .0001 | χ² = 35.1, RR = 0.48 [0.37, 0.62], p < .0001 |
| – Null-subject | 1.19% (8/644) | 5.03% (17/338) | 1.3% (14/1076) | χ² = 13.8, RR = 4.24 [1.83, 9.79], p < .001 | χ² = 0.04, RR = 1.10 [0.46, 2.61], n.s. |
| First-person "I/me/my" /1k w (contrast) | 10.13 (M) | 9.84 (M) | 70.14 (M) | t = 0.18, d = −0.01 [−0.14, +0.12], p = .86 (n.s.) | t ≈ 36, d ≈ +1.10, p < .0001 |
| ***Worker petitions and supplications*** | | | | | |
| Any company-directed petition, % of texts | 6.1% (41/644) | 17.5% (59/338) | 1.5% (16/1076) | χ² = 32.8, RR = 2.87 [1.97, 4.18], p < .0001, φ = 0.18 | χ² = 27.4, RR = 0.24 [0.14, 0.43], p < .0001 |
| – Financial | 3.9% (26/644) | 10.1% (34/338) | 0.09% (1/1076) | χ² = 15.1, RR = 2.61 [1.60, 4.27], p < .001 | χ² = 21.6, RR = 0.024 [0.003, 0.18], p < .0001 |
| – Algorithmic-financial | 1.8% (12/644) | 5.6% (19/338) | 0.0% (0/1076) | χ² = 11.2, RR = 3.16 [1.56, 6.40], p < .001 | absent |
| – Humane treatment | 1.2% (8/644) | 5.3% (18/338) | 0.0% (0/1076) | χ² = 16.1, RR = 4.49 [1.97, 10.2], p < .0001 | absent |
| Any supplication marker | 3.1% (21/644) | 7.7% (26/338) | 0.65% (7/1076) | χ² = 10.7, RR = 2.47 [1.41, 4.34], p < .01, φ = 0.10 | χ² = 16.7, RR = 0.21 [0.09, 0.49], p < .001 |
| – "please" | 1.04% (7/644) | 4.44% (15/338) | 0.65% (7/1076) | χ² = 12.7, RR = 4.28 [1.76, 10.4], p < .001 | χ² = 0.74, RR = 0.62 [0.22, 1.78], n.s. |
| – "if you can" | 0.15% (1/644) | 0.89% (3/338) | 0.0% (0/1076) | χ² = 2.88, RR = 5.99 [0.62, 57.4], p = .09 | absent |
| Ultimatum / exit-threat | 0.59% (4/644) | 1.78% (6/338) | 0.2% (2/1076) | χ² = 3.18, RR = 3.00 [0.85, 10.6], p = .07 | χ² = 1.91, RR = 0.34 [0.06, 1.83], n.s. |
| ***Compulsion and obsession (the Reddit signature)*** | | | | | |
| Compulsion lexicon, % of texts | 0.1% (1/644) | 0.9% (3/338) | 12.7% (137/1076) | χ² = 3.18, RR ≈ 9, p = .07 (small counts) | χ² = 89.5, RR = 85.7 [12.0, 614], p < .0001 |
| Broader obsession / can't-stop | 0.7% (5/644) | 1.8% (6/338) | 14.1% (152/1076) | χ² = 2.18, RR = 2.6, n.s. | χ² = 102, RR = 19.1 [7.86, 46.3], p < .0001 |
| – Self-naming as addicted | 0.0% (0/644) | 0.0% (0/338) | 11.5% (124/1076) | absent in both | corpus-exclusive to Reddit |
| ***Register and stylistic markers*** | | | | | |
| Mean text length (words) | 38 (M) | 75 (M) | 36 (M) | t = 5.71, d = +0.48 [+0.35, +0.62], p < .0001 | t = 0.94, d = −0.05 [−0.15, +0.05], p = .35 (n.s.) |
| Questions per 1,000 words | 0.20 (M) | 1.18 (M) | 59.66 (M) | t = 2.57, d = +0.22 [+0.09, +0.35], p = .01 | t ≈ 60, d = +0.20 (~300×), p < .0001 |
| Ellipses (…), % of texts ≥ 1 | 1.48% (10/644) | 5.03% (17/338) | 7.35% (79/1076) | χ² = 10.0, RR = 3.39 [1.55, 7.44], p < .01, φ = 0.10 | χ² = 30.8, RR = 4.96 [2.59, 9.51], p < .0001 |

***Note.*** *Categorical measures reported as % (n / N). χ² values are Yates-corrected for cell counts < 5. Risk ratios with 95% CIs computed on log scale. All p-values shown are Benjamini–Hochberg FDR-corrected; q-threshold = .05. Cross-corpus comparisons involving Reddit are interpreted descriptively rather than as controlled contrasts since the corpora differ in register (employer-directed adversarial vs peer-confessional).*
